# Association between unmet medication needs after hospital discharge and readmission or death among acute respiratory failure survivors: the addressing post-intensive care syndrome (APICS-01) multicenter prospective cohort study

**DOI:** 10.1186/s13054-021-03848-3

**Published:** 2022-01-07

**Authors:** Samuel M. Brown, Victor D. Dinglas, Narjes Akhlaghi, Somnath Bose, Valerie Banner-Goodspeed, Sarah Beesley, Danielle Groat, Tom Greene, Ramona O. Hopkins, Mustafa Mir-Kasimov, Carla M. Sevin, Alison E. Turnbull, James C. Jackson, Dale M. Needham, for the APICS-01 Study Team, Elise Caraker, Sai Phani Sree Cherukuri, Naga Preethi Kadiri, Tejaswi Kalva, Mounica Koneru, Pooja Kota, Emma Maelian Lee, Mazin Ali Mahmoud, Albahi Malik, Roozbeh Nikooie, Darin Roberts, Sriharsha Singu, Parvaneh Vaziri, Katie Brown, Austin Daw, Mardee Merrill, Rilee Smith, Ellie Hirshberg, Jorie Butler, Benjamin Hoenig, Maria Karamourtopoulos, Margaret Hays, Rebecca Abel, Craig High, Emily Beck, Brent Armbruster, Darrin Applegate, Melissa Fergus, Naresh Kumar, Megan Roth, Susan Mogan, Rebecca Abel, Andrea De Souza Licht, Isabel Londono, Julia Larson, Krystal Capers, Maria Karamourtopoulos, Benjamin Hoenig, Andrew Toksoz-Exley, Julia Crane

**Affiliations:** 1 Pulmonary and Critical Care Medicine, Intermountain Medical Center, Salt Lake City, UT USA; 2 Pulmonary and Critical Care Medicine, University of Utah, Salt Lake City, UT USA; 3 Center for Humanizing Critical Care, Intermountain Medical Center, Salt Lake City, UT USA; 4 Outcomes After Critical Illness and Surgery (OACIS) Group and Pulmonary and Critical Care Medicine, School of Medicine, Johns Hopkins University, Baltimore, MD USA; 5 Department of Internal Medicine, Yale School of Medicine, New Haven, CT USA; 6 Beth Israel Deaconess Medical Center, Boston, MA USA; 7 Biostatistics and Epidemiology, University of Utah, Salt Lake City, UT USA; 8 Psychology Department and Neuroscience Center, Brigham Young University, Provo, UT USA; 9 Salt Lake City Veterans Administration, Salt Lake City, UT USA; 10 Vanderbilt University Medical Center, Nashville, TN USA; 11 Shock Trauma ICU, Intermountain Medical Center, 5121 S. Cottonwood Street, Murray, UT 84107 USA

**Keywords:** Acute respiratory failure, Long-term outcomes, Discharge planning, Health services research

## Abstract

**Introduction:**

Survivors of acute respiratory failure (ARF) commonly experience long-lasting physical, cognitive, and/or mental health impairments. Unmet medication needs occurring immediately after hospital discharge may have an important effect on subsequent recovery.

**Methods and analysis:**

In this multicenter prospective cohort study, we enrolled ARF survivors who were discharged directly home from their acute care hospitalization. The primary exposure was *unmet medication needs*. The primary outcome was hospital readmission or death within 3 months after discharge. We performed a propensity score analysis, using inverse probability weighting for the primary exposure, to evaluate the exposure–outcome association, with an a priori sample size of 200 ARF survivors.

**Results:**

We enrolled 200 ARF survivors, of whom 107 (53%) were female and 77 (39%) were people of color. Median (IQR) age was 55 (43–66) years, APACHE II score 20 (15–26) points, and hospital length of stay 14 (9–21) days. Of the 200 participants, 195 (98%) were in the analytic cohort. One hundred fourteen (57%) patients had at least one unmet medication need; the proportion of medication needs that were unmet was 6% (0–15%). Fifty-six (29%) patients were readmitted or died by 3 months; 10 (5%) died within 3 months. Unmet needs were not associated (risk ratio 1.25; 95% CI 0.75–2.1) with hospital readmission or death, although a higher proportion of unmet needs may have been associated with increased hospital readmission (risk ratio 1.7; 95% CI 0.96–3.1) and decreased mortality (risk ratio 0.13; 95% CI 0.02–0.99).

**Discussion:**

Unmet medication needs are common among survivors of acute respiratory failure shortly after discharge home. The association of unmet medication needs with 3-month readmission and mortality is complex and requires additional investigation to inform clinical trials of interventions to reduce unmet medication needs.

*Study registration number*: NCT03738774. The study was prospectively registered before enrollment of the first patient.

**Supplementary Information:**

The online version contains supplementary material available at 10.1186/s13054-021-03848-3.

## Introduction

Post-discharge survival for patients with acute respiratory failure (ARF) is increasing [1–5]. Survivors often suffer from long-lasting impairments in physical, cognitive, and/or mental health status, and face substantial financial burden due to delayed return to work and associated loss of earnings, healthcare costs, and changes in insurance [6–20]. Survivors often experience fragmented healthcare after hospital discharge and mismatches between the healthcare services needed and those received during the vulnerable weeks immediately after discharge [21]. The immediate post-discharge period may be a time of particular vulnerability, with a potential outsize impact on subsequent recovery. Readmissions are common, expensive, and potentially avoidable [22–24]. Retrospective cohorts based on claims data have suggested that failures to follow through may be associated with early readmissions [25–27]. Similar to post-discharge management of heart failure [28], medications represent a particularly important target in the post-discharge care of patients who have survived an ICU admission [29–32]. Limited prospective data exist to clarify the relationships among support and care early after hospital discharge and subsequent clinical outcomes. We thus undertook a multicenter prospective cohort study to explore the associations of unmet medication needs after hospital discharge and hospital readmission or death 3 months after discharge among survivors of acute respiratory failure who were discharged directly to home. Early results have been published in abstract form [33, 34]. We have previously reported the underlying rationale for the study [35].

## Methods and analysis

### Study design

This is a prospective multicenter cohort study of ARF survivors conducted at six hospitals affiliated with five US medical centers. We have published the methods in detail previously [36]; we summarize them here, with the full study protocol and statistical analysis plan provided in the Online Supplement. This paper reports our analysis of unmet medication needs, as distinct from other types of healthcare needs, which will be analyzed and reported subsequently.

### Patients

We enrolled patients with ARF who were being discharged to home directly from the acute care hospital. We defined ARF as ≥ 24 consecutive hours of (1) mechanical ventilation via an endotracheal tube, (2) noninvasive ventilation, or (3) high-flow nasal cannula with FIO_2_ ≥ 0.5 and flow rate ≥ 30 L/min. We excluded patients whose respiratory support was provided for reasons other than respiratory failure (e.g., airway obstruction or coma), for whom telephone-based follow-up was not feasible, or with preexisting dementia. Dementia was defined using Informant Questionnaire on Cognitive Decline in the Elderly (IQ-CODE) [37] screening, as commonly used in other ARF follow-up studies [13, 38].

### Primary exposure

*Unmet medication needs* were the primary exposure of interest, assessed as soon as possible within a time window of 7–28 days after hospital discharge. Unmet medication needs were measured using a subset of a larger instrument we developed. Details of this larger instrument are provided in the online supplemental methods and have been published previously [36]. In this report, we focus on medication-related needs given the immediate relevance of medication needs (as opposed to non-medication interventions that may not be scheduled until after initial follow-up has been completed).

Immediately after discharge, the medication needs instrument was completed. The initial telephone call to ascertain the primary exposure (unmet medication needs) was performed after a grace period of 7 days (to allow time for medical needs to be met), with the time window extending for up to 21 additional days. During the initial call, the medication needs instrument was used to identify and categorize each discharge need as completed or not completed based on changes in medication prescriptions (whether to start a new medicine, stop an established medicine, or change a medication dose). In other words, the total number of medication instructions/orders was the denominator. The numerator was the number of times an instruction was not implemented in the early post-discharge period. Thus, an unmet need might be, e.g., failure to start a new medication or failure to stop a medication that had been discontinued. The prespecified primary exposure variable was the proportion of medication needs that were unmet (e.g., if a patient had 8 needs and 2 were not met, the proportion unmet would be 25%). For purposes of analysis, this proportion was treated as above versus below the median proportion for the overall cohort.

### Primary outcome

The prespecified primary outcome was a composite binary outcome of hospital readmission or death within 3 months of discharge to home from the index hospitalization.

### Secondary outcomes

Secondary outcomes included the constituent elements of the composite primary outcome as well as additional outcomes measured during 3- and 6-month centralized telephone-based follow-up assessments, including: at 3 months: cognitive impairment, depression, anxiety, post-traumatic stress disorder-related symptoms, emergency department visits and other healthcare utilization, and at 6 months: mortality, healthcare utilization, health-related quality of life, and return to work. This assessment included the recommended Core Outcome Set (COS) and associated measurement instruments for ARF survivors (see www.ImproveLTO.com) [39, 40]. The centralized follow-up process followed established practices for optimizing participant retention [41–45].

### Statistical analysis

Full details of the analysis are presented in the Statistical Analysis Plan provided in the Online Supplement. The primary analyses were specified before review of any data. The primary research question was whether unmet medication needs shortly after hospital discharge to home are associated with readmission or death within 3 months of hospital discharge in ARF survivors. The ultimate inferential target was whether meeting medication needs in the early post-discharge period will decrease hospital readmission or death. The primary analysis compared patients with an above-median proportion of unmet medication needs to patients with a below-median proportion of unmet medication needs.

Key methodological considerations for evaluating the association of the primary exposure and outcome are the management of reverse causation (the disease process underlying readmission or death by 3 months is the reason why the medication needs are unmet) and confounding by indication (e.g., more complex discharge plans, with a higher risk for unmet needs, occur for patients at higher risk for death or readmission). We used covariate balancing propensity score (CBPS) [46] adjustment to estimate the average causal (“treatment”) effects in the treated. Under the assumption of no residual confounding, the respective treatment effects are the average reduction in the risk of the primary outcome among patients with above-median levels of unmet medication needs if an intervention reduced their proportion of unmet needs to below the median. (This dichotomization was used to improve the stability of regression models.) Secondary analyses addressed the proportions of unmet needs as continuous rather than dichotomized variables.

Covariates for multivariable models included patient characteristics at baseline and hospital discharge, including demographics, tobacco and alcohol use, severity of illness measures, and hospital length of stay. Covariates were specified before data were reviewed. The primary outcome model included enrolling site, age, and sex, and number of post-discharge medication needs as prespecified covariates. After finalizing the propensity score model, we used Poisson regression to estimate the risk ratio comparing the risk of the primary outcome between the two groups (those with above- vs. below-median proportion of unmet medication needs).

#### Sample size and power

Assuming a post-discharge attrition rate of 24%, enrollment of 200 patients would result in 152 patients in the analytic cohort. We anticipated that 35% of patients would die or be readmitted by 3 months [30, 47]. Using statistical simulation, we estimated 80% power with two-sided *α* = 0.05 to detect an increase in the risk of the composite outcome from 30% for those in the lower unmet needs category to 53% for those in higher unmet needs category.

#### Ethical considerations

This study was funded by the U.S. Department of Defense (grant # W81XWH-18-1-0813) and was registered at ClinicalTrials.gov (NCT03738774) before enrollment of the first patient. The study received approval from, and was overseen by, the central Institutional Review Board (IRB) at Vanderbilt University with additional oversight by the Human Research Protections Office (HRPO) of the Department of Defense. According to local policy, the Veterans Affairs hospital site was overseen by its designated IRB at the University of Utah. The study was fully approved before enrollment of the first patient. Informed consent was obtained prospectively from all participants or their legally authorized representatives.

## Results

### Patients

**Fig. 1:**
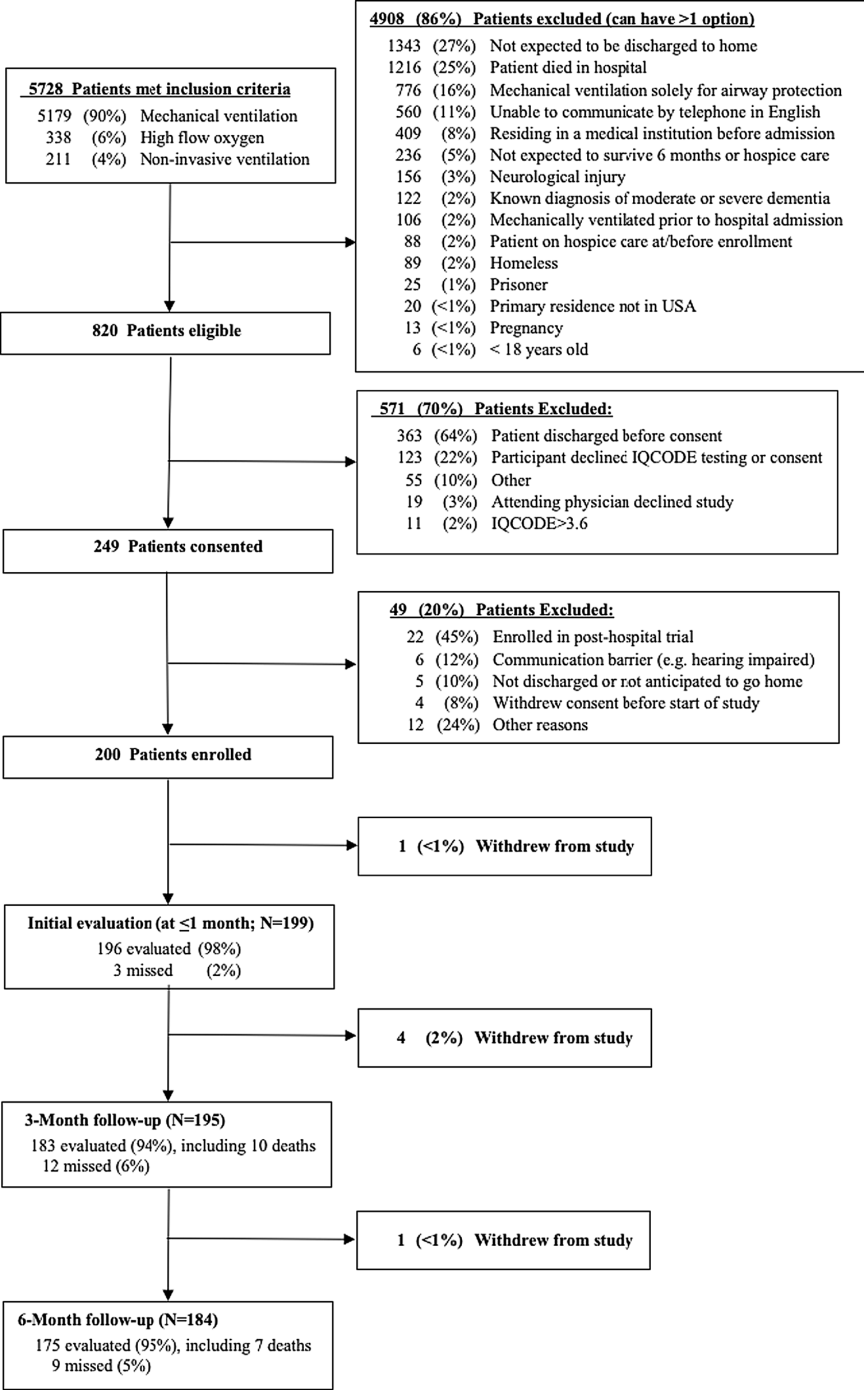
Flow diagram of patient participation in APICS-01

We enrolled patients from January 2019 to August 2020. Among 5728 patients meeting inclusion criteria, 820 met all eligibility criteria. The most common reason for exclusion was for patients not being discharged directly home. Two hundred patients were consented and enrolled in the study (Fig. 1). After one patient withdrew prior to study procedures, we achieved 98% (196/199) follow-up for ascertainment of primary exposure; we ascertained the primary endpoint at 3 months in 183/195 (94%) of eligible patients (4 additional patients withdrew) and identified the primary endpoint from medical record review in another 12 patients. The analytic cohort thus contained 195 patients in whom both the primary exposure and outcome were known.

Baseline characteristics of enrolled patients are presented in Table 1. Median age was 55 (IQR 43–66) years, 104 (53%) were female, and 77 (39%) were people of color. Median duration of ICU and hospital stay was 6 (IQR 4–10) and 14 (IQR 9–21) days, respectively (Additional file 1: Table S1). At hospital discharge, 50 (26%) patients were oxygen dependent and 11 (6%) dialysis dependent (Additional file 1: Table S2).

**Table 1:** Baseline characteristics of analyzed patients (*n* = 195)

Attribute	Central tendency (dispersion)
Age, years, median (IQR)	55 (43–66)
Female sex, *n* (%)	104 (53.3%)
Race, *n* (%)	
Black	48 (24.6%)
Hispanic/Latinx	5 (2.6%)
Non-Hispanic White	129 (66.2%)
Other/multiple	13 (6.7%)
Body mass index (kg/m^2^), median (IQR)*	29.5 (24.7–36.5)
Respiratory support at enrollment, *n* (%)	
Invasive mechanical ventilation	145 (74.4%)
Non-invasive mechanical ventilation	13 (6.7%)
High-flow nasal cannula	37 (19.0%)
Transferred from outside hospital, *n* (%)	75 (38.5%)
APACHE II, score, median (IQR)	20 (15–26)
Current smoker, *n* (%)	31 (15.9%)
Acute respiratory distress syndrome, *n* (%)	61 (31.3%)
Clinical frailty scale, median (IQR)	3 (2–4)
Multidimensional scale perceived social support, median (IQR)	72 (60–81)
Alcohol use disorders identification test (AUDIT), median (IQR)	1 (0–2)
Abnormal AUDIT, *n* (%)	16 (8.2%)
Functional capacity index, median (IQR)	2 (1–3)
Charlson comorbidity index, median (IQR)	1 (0–3)
Resides at home before admission	190 (97.4%)
Employment status prior to admission (*n* = 161)	
Working (full/part-time), looking for work, or in school	85 (43.6%)
Unemployed, not looking for work	7 (3.6%)
Retired	29 (14.9%)
Receiving disability payments	32 (16.4%)
Prior COVID-19 hospitalization, *n* (%)	0 (0.0%)
Tested positive for COVID-19 during admission, *n* (%)	33 (16.9%)

^*^BMI missing for 13 participants. Multivariate imputation by chained equations with baseline and discharge variables was used to impute missing values

At hospital discharge to home, the median number of total medication needs (i.e., the denominator for calculating the proportion of unmet medication needs) per patient was 13 (IQR 9–19). Overall, 114 (58%) patients had at least one unmet medication need; the median (IQR) proportion of medication needs that were unmet was 6% (0–15%). In Table 2, we display the differences in baseline characteristics between those above versus below the median proportion of unmet medication needs. Patients above (vs. below) the median number of unmet medication needs more commonly were non-Hispanic White, transferred to the study hospital from a referring hospital, a current smoker, and had ARDS during the index hospitalization (Table 2). Adjustment for propensity for the primary exposure yielded good covariate balance (Additional file 1: Table S3 and Additional file 1: Fig. S1).

**Table 2:** Baseline characteristics of enrolled patients divided by high versus low unmet medication needs

Attribute	Unmet needs < 0.06, *n* = 97	Unmet needs ≥ 0.06, *n* = 98
Age, years, median (IQR)	55 (41–66)	55 (44.2–63.8)
Female sex, *n* (%)	47 (48.5%)	57 (58.2%)
Race, *n* (%)		
Black	34 (35.1%)	14 (14.3%)
Latinx	2 (2.1%)	3 (3.1%)
Non-Hispanic White	54 (55.7%)	75 (76.5%)
Other/multiple	7 (7.2%)	6 (6.1%)
Body mass index (kg/m^2^), median (IQR)	28.6 (24.5–34.9)	29.8 (24.9–37.6)
Respiratory support at enrollment, *n* (%)		
Invasive mechanical ventilation	72 (74.2%)	73 (74.5%)
Non-invasive mechanical ventilation	7 (7.2%)	6 (6.1%)
High-flow nasal cannula	18 (18.6%)	19 (19.4%)
Transferred from outside hospital, *n* (%)	33 (34.0%)	42 (42.9%)
APACHE II, score, median (IQR)	21 (16–26)	20 (14–26)
Current smoker, *n* (%)	13 (13.4%)	18 (18.4%)
Insurance, *n* (%)		
Private and public	18 (18.6%)	6 (6.1%)
Private	42 (43.3%)	52 (53.1%)
Public	33 (34.0%)	33 (33.7%)
None/other	4 (4.1%)	7 (7.1%)
Acute respiratory distress syndrome, *n* (%)	29 (29.9%)	32 (32.7%)
Clinically frailty scale, median (IQR)	3 (2–4)	3 (2–4)
Multidimensional scale perceived social support, median (IQR)	72 (63–80)	72 (59.2–81)
AUDIT score, median (IQR)	0 (0–2)	1 (0–2)
Functional capacity index, median (IQR)	2 (1–3)	2 (1–3)
Charlson comorbidity index, median (IQR)	2 (0–3)	1 (0–2.8)
Resides at home before admission, *n* (%)	93 (95.9%)	97 (99.0%)
Tested positive for COVID-19 during admission, *n* (%)	14 (14.4%)	19 (19.4%)
Hospital length of stay, days, median (IQR)	14 (9–24)	14 (9.2–19)

### Study outcomes

**Fig. 2:**
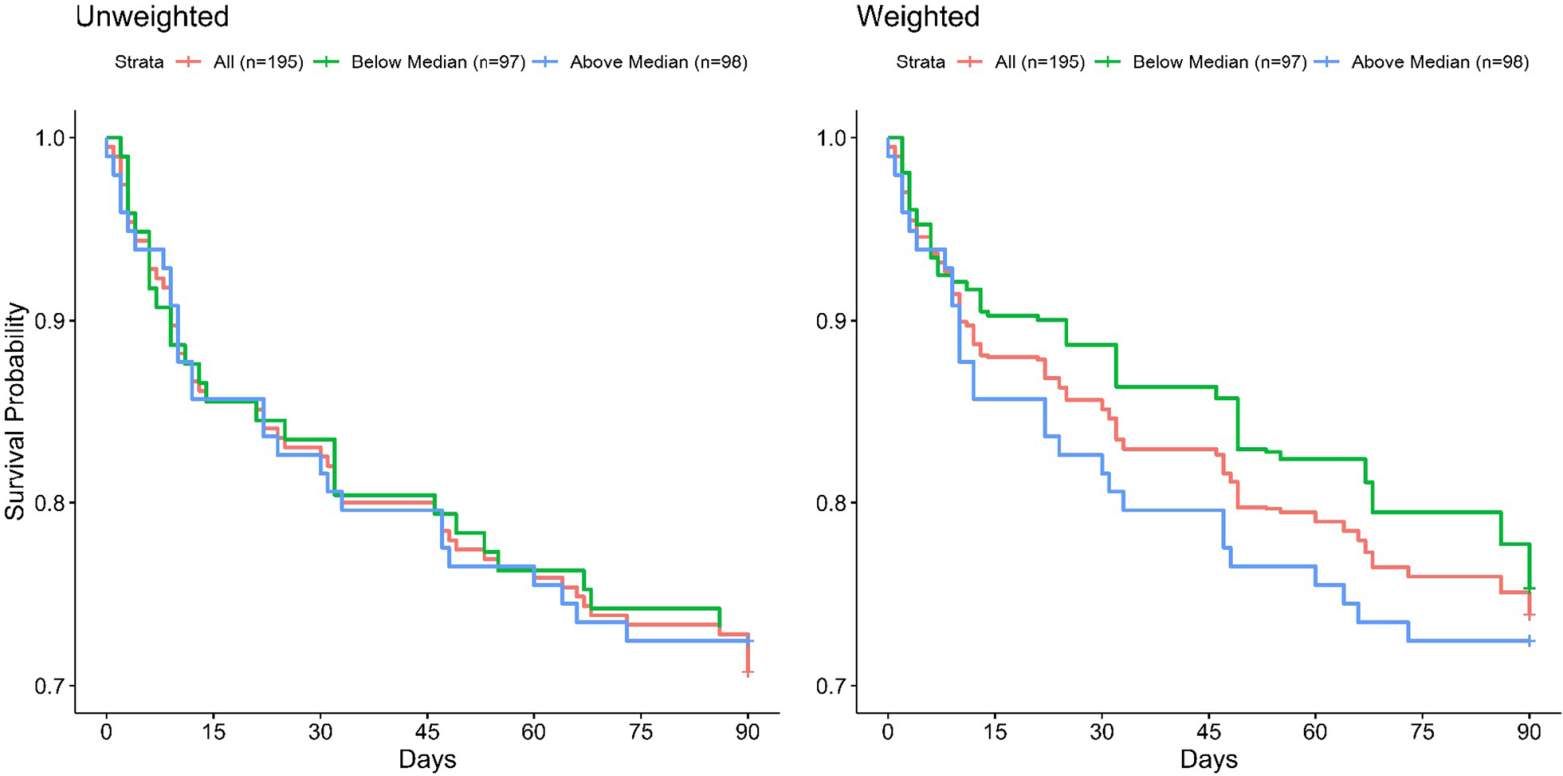
Kaplan–Meier curves for combined readmission and mortality; grouped by proportion of unmet medication needs

Overall, within 3 months, 56 (29%) patients died or were readmitted to hospital, with 10 (5%) dead and 52 (27%) readmitted. Median time to death or readmission was 14 (IQR 7–47) days (Table 3). Causes of death are presented in Additional file 1: Table S4. Kaplan–Meier curves for the overall cohort and for patients above vs. below the median proportion of unmet medication needs are shown in Fig. 2, Additional file 1: Figs. S2 and S3. At 3 months (Additional file 1: Table S5) the median (IQR) health utility from the EQ-5D-5L was 0.8 (0.7–1.0), with one quarter of patients meeting criteria for anxiety, one quarter meeting criteria for depression, and 11% had post-traumatic stress symptoms. One-third of patients had evidence for cognitive impairment. By 6-month follow-up (Additional file 1: Tables S6 and S7), 17 (9%) patients had died.

**Table 3:** Primary outcome and constituents at 3 months, *n* = 195

Attribute	Central tendency (dispersion)
No death or readmission before 3 months, *n* (%)	139 (71.3%)
Death or readmission before 3 months, *n* (%)	56 (28.7%)
Time to primary outcome among those achieving primary outcome, days, median, (IQR)	14 (7–47)
Death before 3 months, *n* (%)*	10 (5.1%)
Time to death among decedent, days, median (IQR)	70.5 (36.3–80)
Hospital readmission before 3 months, *n* (%)	52 (26.7%)
Time to readmission among those readmitted, days, median (IQR)	12 (6–32.75)

^*^Death before 3-month follow-up includes 6 participants that also were readmitted before the 3-month follow-up

### Primary analysis

The primary regression analysis did not identify a statistically significant association (risk ratio 1.25, 0.75–2.1) between above (vs. below) median unmet medication needs and the primary endpoint—death or readmission within 3 months (Additional file 1: Table S8). Regression models of the constituent endpoints were also performed. The risk ratio for above (vs. below) median proportion of unmet medication needs for death was 0.13 (0.02–0.99; *p* = 0.05), while the risk ratio for readmission was 1.7 (0.96–3.1; *p* = 0.07) (Additional file 1: Table S9). On sensitivity analyses, treating the proportion of unmet needs as a continuous variable yielded similar results (Additional file 1: Table S10), as did adjusting for time (after hospital discharge) to assessment of unmet medication needs (Additional file 1: Table S11). Dichotomizing the exposure into all medication needs met versus any medication need unmet yielded similar results, with 95% confidence intervals that clearly excluded unity (Additional file 1: Table S12).

The discordance in associations between unmet medication needs and the risks of mortality and of readmission was evaluated in exploratory analyses. For participants who died, key unmet medication needs were primarily related to mental health medications (anti-depressants and anxiolytics), other prescription medications (narcotics, muscle relaxants), and new prescriptions for corticosteroids and anticoagulants. Two of 10 patients died from cancer, with the other causes of death among the other 8 decedents being heterogenous (Additional file 1: Table S4). The patients who died had longer hospital and ICU length of stay, greater baseline frailty, were more likely to be enrolled into a discharge navigator program, and to have higher numbers of medication needs (16 vs. 12 needs) at the time of hospital discharge.

## Discussion

This multicenter prospective cohort study of survivors of ARF found that more than half of participants had unmet medication needs shortly after discharge. The relationship between these unmet medication needs and 3-month outcomes appears complex: propensity-adjusted regression analysis found that having fewer unmet medication needs may have been associated with a higher risk of mortality and a lower risk of hospital readmission, leading to an apparent lack of association with the primary composite outcome (death or readmission). We hypothesize that for patients who died, having medication needs met was a marker of residual confounding by indication—sicker patients (i.e., those more likely to die) were more likely to have close attention paid to their discharge plans. These findings suggest that interventions purely targeted at assuring discharge medication needs are met may oversimplify the complex relationships among medication needs and patient outcomes.

Our data suggest that ARF survivors have high numbers of medication needs and more than half have at least one unmet medication need. This evidence is consistent with other observations about polypharmacy in this population [31, 32]. Notably, our study’s secondary outcomes, at 3-month and 6-month follow-up, were consistent with prior cohorts noting high rates of psychological and cognitive disability after discharge [48, 49].

Our findings point toward the importance of additional research to design and test optimal interventions to improve recovery among acute respiratory failure survivors. These observations add to concerns raised about readmission-reduction programs intended to improve compliance with government metrics which led to worse patient outcomes overall [50–55] and emphasize the need for evidence from randomized controlled trials to support interventions and programs designed to improve post-discharge outcomes.

Strengths of this study include its multicenter recruitment and robust follow-up rates (~ 95%). While this was not a randomized trial, we prospectively enrolled patients and employed appropriate statistical methods to estimate the average treatment effect. Limitations of the study include lack of randomization, lack of data on medications patients were receiving at baseline, lack of specific data regarding the reasons that medication needs were unmet, and no in-depth data on palliative care interactions. We also acknowledge that small numbers of deaths severely limit the certainty of the association between unmet medication needs and mortality. Although study sites perform medication reconciliation routinely at hospital discharge, we did not perform a separate confirmation that the discharge medication plans were optimal.

The findings of the APICS-01 study will inform ongoing work to understand and test optimal approaches to supporting survivors of ARF. Substantial prior work has focused on ICU aftercare and recovery clinics, which attempt to provide and/or coordinate within one clinic, a range of healthcare-related services. Existing data on the effectiveness of these clinics have been mixed [35, 56–58], perhaps reflecting the generally late (e.g., 3 months after discharge) initiation of such services. This model of support may miss an early window of vulnerability among ARF survivors, as suggested by a pilot randomized trial [47]. The pragmatic IMPACTS trial also suggested that a nurse navigator might improve outcomes among sepsis survivors [59]. The IMPACTS intervention included symptom monitoring, medication review, and targeted palliative care referrals. The present study provides confirmatory evidence that the early post-discharge period is an important target for interventions to improve outcomes for ARF survivors.

## Conclusion

The APICS-01 study expands on prior retrospective, single-center or single-system observations via a multicenter, multisystem prospective cohort study, demonstrating that ARF survivors are at high risk for early post-discharge readmission and death. Moreover, ARF survivors, upon discharge to home, have many medication prescriptions and changes in medications. Distinct processes of unmet medication needs may separately drive early death and readmission. These findings are crucial to inform the design and evaluation of interventions to improve outcomes for this high-needs patient population.

## Supplementary Information

**Additional file 1:** Online supplemental materials.

## Data Availability

In order to protect patient privacy and comply with relevant regulations, identified data are unavailable. Requests for deidentified data from qualified researchers with appropriate ethics board approvals and relevant data use agreements will be processed by the Intermountain Office of Research, officeofresearch@imail.org.
