# Supplementary material for: Association between unmet medication needs after hospital discharge and readmission or death among acute respiratory failure survivors: the addressing post-intensive care syndrome (APICS-01) multicenter prospective cohort study
Source: Crit Care. 2022 Jan 7;26:6. doi: 10.1186/s13054-021-03848-3 (PMC8738999; doi:10.1186/s13054-021-03848-3)
Supplement: Supplementary file 1 — Additional file 1: Online supplemental materials. [file 13054_2021_3848_MOESM1_ESM.docx]

ONLINE SUPPLEMENT

Association between unmet medication needs after hospital discharge and re-admission or death among acute respiratory failure survivors: the Addressing Post-Intensive Care Syndrome (APICS-01) multicenter prospective cohort study

Samuel M. Brown MD MS^1,2,3^ , Victor D. Dinglas, MPH^4^, Narjes Akhlaghi, MD MPH^5^, Somnath Bose, MD^6^, Valerie Banner-Goodspeed, MPH^6^, Sarah Beesley, MD MS^1,2,3^, Danielle Groat, PhD^1,3^, Tom Greene^7^, PhD, Ramona O. Hopkins, PhD^3,8^, Mustafa Mir-Kasimov, MD^2,9^, Carla M. Sevin, MD^10^, Alison E. Turnbull, DVM, MPH, PhD^4^, James C. Jackson, PsyD^10^, Dale M Needham, FCPA MD PhD^4^, for the APICS-01 Study Team

^1^Pulmonary and Critical Care Medicine, Intermountain Medical Center, Salt Lake City, UT USA

^2^Pulmonary and Critical Care Medicine, University of Utah, Salt Lake City, UT USA

^3^Center for Humanizing Critical Care, Intermountain Medical Center, Salt Lake City, UT USA

^4^Outcomes After Critical Illness and Surgery (OACIS) Group and Pulmonary and Critical Care Medicine, School of Medicine, Johns Hopkins University, Baltimore, MD USA

^5^Department of Internal Medicine, Yale School of Medicine, New Haven, CT USA

^6^Beth Israel Deaconess Medical Center, Boston, MA USA

^7^Biostatistics and Epidemiology, University of Utah, Salt Lake City, UT USA

^8^Psychology Department and Neuroscience Center, Brigham Young University, Provo, UT USA

^9^Salt Lake City Veterans Administration, Salt Lake City, UT USA

^10^Vanderbilt University Medical Center, Nashville, TN USA

[Additional file 1: Methods 4](#_Toc87891704)

[Additional file 1: Results -- Figures 5](#_Toc87891705)

Additional file 1: [Figure S1: Covariate balance of observed and adjusted characteristics based on unmet medication needs 5](#_Toc87891706)

Additional file 1: [Figure S2. Kaplan-Meier curves for survival without readmission, by above vs. below median number of unmet medication needs 6](#_Toc87891707)

Additional file 1: [Figure S3. Kaplan-Meier curves for survival, by above vs. below median number of unmet medication needs 6](#_Toc87891708)

[Additional file 1: Results – Tables 7](#_Toc87891709)

Additional file 1: [Table S1: Pre-discharge attributes of enrolled patients 7](#_Toc87891710)

Additional file 1: [Table S2: Status at hospital discharge 7](#_Toc87891711)

Additional file 1: [Table S3: Distribution of baseline characteristics after unmet medication needs propensity adjustment, including measures for balance: standardized mean differences (SMD), and Kolmogorov-Smirnov statistic (KS) 7](#_Toc87891712)

Additional file 1: [Table S4: Cause of death at 3-month follow-up 9](#_Toc87891713)

Additional file 1: [Table S5: Descriptive statistics of secondary outcomes at 3-month follow-up (n = 194) 9](#_Toc87891714)

Additional file 1: [Table S6: Mortality at 6 months, n=186 10](#_Toc87891715)

Additional file 1: [Table S7: Descriptive statistics of secondary outcomes at 6-month follow-up (n = 165) 10](#_Toc87891716)

Additional file 1: [Table S8: Primary outcome estimates for exposure and covariates 11](#_Toc87891717)

Additional file 1: [Table S9. Primary outcome estimates for exposure constituents and covariates 11](#_Toc87891718)

Additional file 1: [Table S10. Summary of findings for regression models using unmet needs as a continuous variable 12](#_Toc87891719)

Additional file 1: [Table S11. Sensitivity analysis, includes adjustment for time to assessment of discharge needs follow up 12](#_Toc87891720)

Additional file 1: [Table S12. Sensitivity analysis, medication needs categorized as all met or 1 or more needs unmet 12](#_Toc87891721)

# Additional file 1: Methods

The protocol and statistical analysis plan are attached to the end of this document. The full analysis plan was finalized before review of any data other than the distribution of post-discharge needs among approximately 80 patients, which was necessary to categorize the exposure variables (early descriptive statistics identified dichotomization above or below the median as an appropriate threshold).

The unmet medication needs instrument employed in this study is a subset of a larger unmet healthcare needs instrument. We developed this instrument, and iteratively refined it based on pilot testing across all study sites, to measure healthcare needs, as identified in hospital discharge documentation. Initial development of this instrument was based on published literature and extensive clinical experience at ICU aftercare and recovery clinics at two study site hospitals.(1, 2) Healthcare needs assessed with this instrument include, but are not limited to, durable medical equipment, oxygen, home health services, dialysis, follow-up healthcare appointments, substance use counseling, and medications.(2-4)

# Additional file 1: Results -- Figures

Additional file 1: Figure S1: Covariate balance of observed and adjusted characteristics based on unmet medication needs.


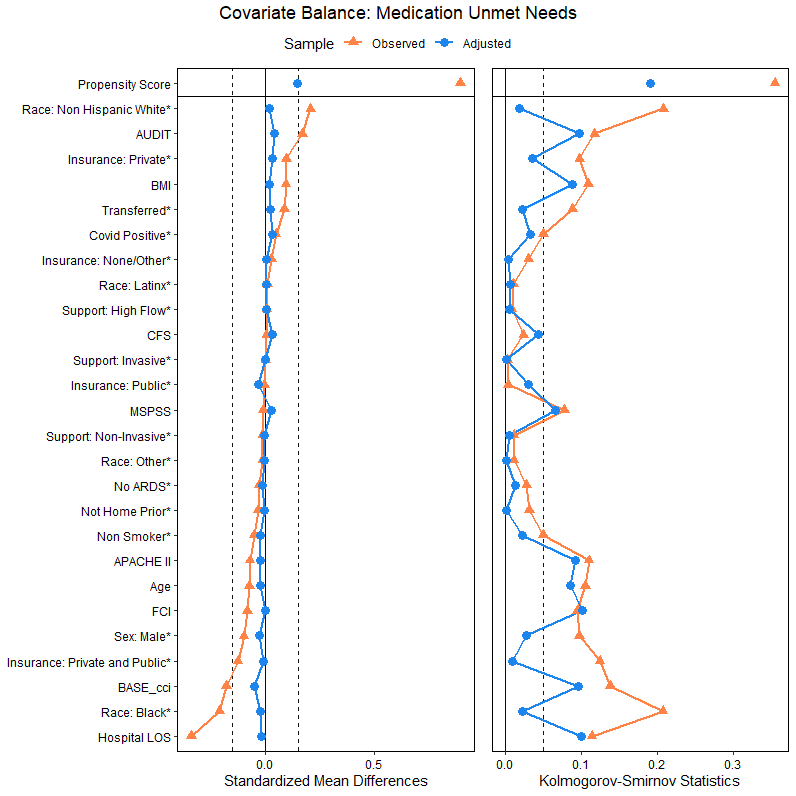


* indicates value is the raw (unstandardized) difference in means

Additional file 1: Figure S2. Kaplan-Meier curves for survival without readmission, by above vs. below median number of unmet medication needs.


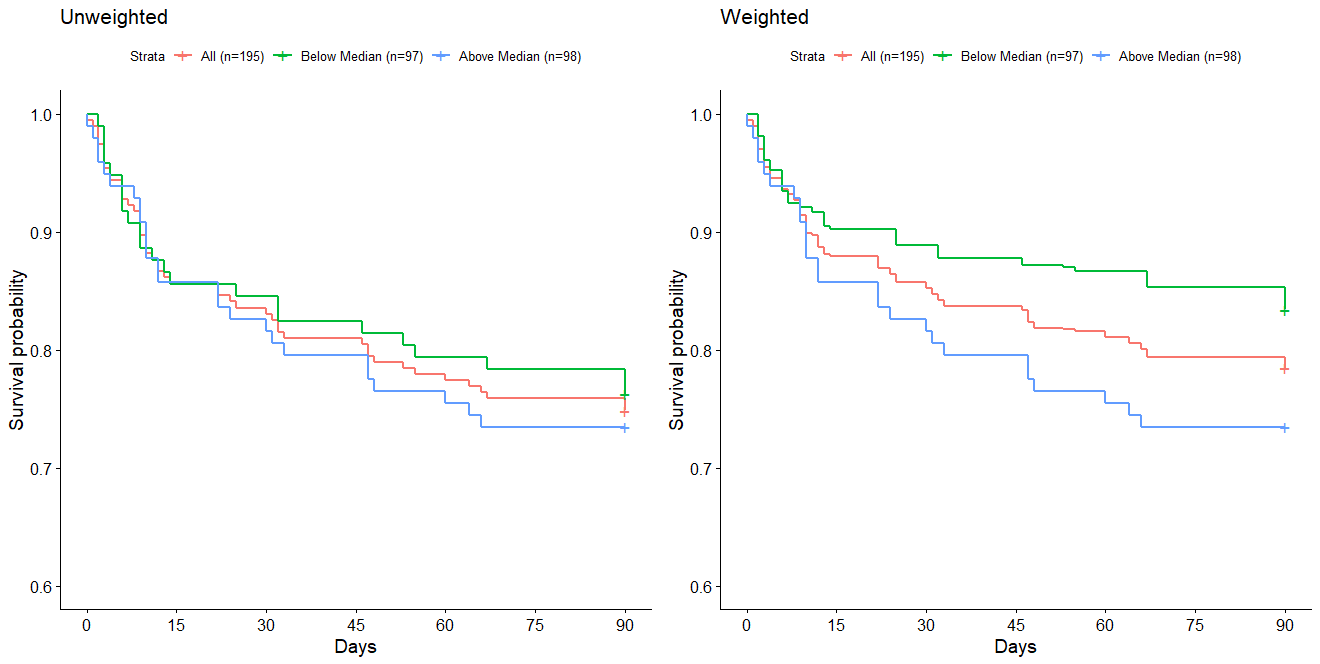


Additional file 1: Figure S3. Kaplan-Meier curves for survival, by above vs. below median number of unmet medication needs.


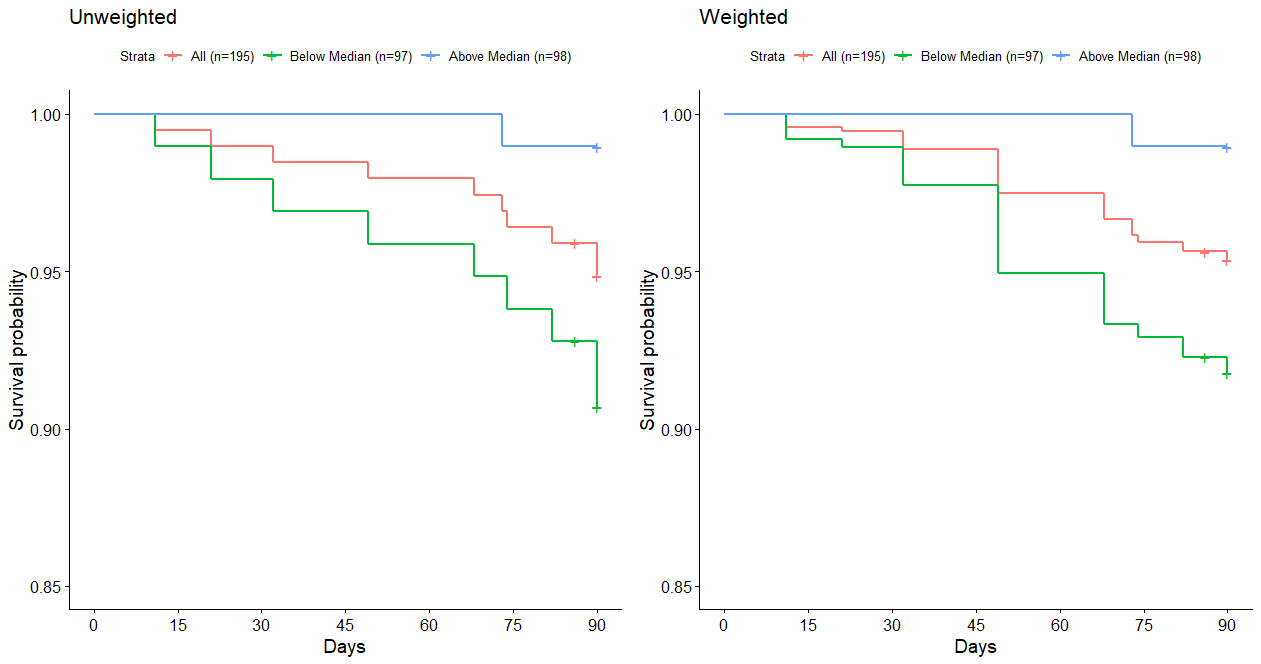


## Additional file 1: Results – Tables

Additional file 1: Table S1: Pre-discharge attributes of enrolled patients.

| Attribute | Central tendency (dispersion) |
| --- | --- |
| ICU LOS (days), median (IQR) | 6 (4-10) |
| Hospital LOS (days), median (IQR) | 14 (9-21) |
| Ever mechanically ventilated, n (%) | 151 (77.4%) |
| Duration of mechanical ventilation (days), median (IQR) | 2.6 (0.8-4.8) |
| Ever delirious, n (%) | 100 (51.3%) |
| Ever dialysis, n (%) | 21 (10.8%) |
| Tracheostomy during ICU stay, n (%) | 8 (4.1%) |
| ECMO during ICU stay, n (%) | 6 (3.1%) |
| ECMO: extracorporeal membrane oxygenation; ICU: intensive care unit; LOS: length of stay | |

Additional file 1: Table S2: Status at hospital discharge.

| Attribute | n (%) |
| --- | --- |
| Dialysis dependent | 11 (5.6%) |
| Vasopressor dependent | 0 (0.0%) |
| Tracheostomy at discharge | 0 (0.0%) |
| Oxygen dependent | 50 (25.6%) |
| Do not resuscitate/intubate orders | 2 (1.0%) |
| Enrolled in navigator program | 32 (16.4%) |

Additional file 1: Table S3: Distribution of baseline characteristics after unmet medication needs propensity adjustment, including measures for balance: standardized mean differences (SMD), and Kolmogorov-Smirnov statistic (KS).

| Attribute | Unmet needs < 0.06 n=97 | Unmet needs >= 0.06 n=98 | SMD | KS |
| --- | --- | --- | --- | --- |
| Age, years, median (IQR) | 48 (21.5-77.9) | 55 (44.3-63.8) | -0.022 | 0.086 |
| Female sex, n (%) | 53.78 (55.4%) | 57 (58.2%) | -0.027 | 0.027 |
| Race, n (%) |  |  |  |  |
| Black | 34 (35.1%) | 14 (14.3%) | -0.023 | 0.023 |
| Latinx | 2 (2.1%) | 3 (3.1%) | 0.006 | 0.006 |
| Non-Hispanic White | 54 (55.7%) | 75 (76.5%) | 0.018 | 0.018 |
| Other/multiple | 7 (7.2%) | 6 (6.1%) | -0.001 | 0.001 |
| Body mass index (kg/m2), median (IQR) | 29.2 (9.4-43.6) | 29.8 (24.9-37.6) | 0.021 | 0.087 |
| Respiratory support at enrollment, n (%) |  |  |  |  |
| Invasive mechanical ventilation | 72 (74.2%) | 73 (74.5%) | 0.001 | 0.001 |
| Non-invasive mechanical ventilation | 7 (7.2%) | 6 (6.1%) | -0.005 | 0.005 |
| High-flow nasal cannula | 18 (18.6%) | 19 (19.4%) | 0.004 | 0.004 |
| Transferred from outside hospital, n (%) | 39.47 (40.7%) | 42 (42.9%) | 0.022 | 0.022 |
| APACHE II, score, median (IQR) | 15.6 (7.9-29.9) | 20 (14-26) | -0.022 | 0.092 |
| Current smoker, n (%) | 15.73 (16.2%) | 18 (18.4%) | -0.021 | 0.021 |
| Insurance, n (%) |  |  |  |  |
| Private and Public | 18 (18.6%) | 6 (6.1%) | -0.009 | 0.009 |
| Private | 42 (43.3%) | 52 (53.1%) | 0.035 | 0.035 |
| Public | 33 (34%) | 33 (33.7%) | -0.030 | 0.030 |
| None/Other | 4 (4.1%) | 7 (7.1%) | 0.004 | 0.004 |
| Acute Respiratory Distress Syndrome, n (%) | 30.46 (31.4%) | 32 (32.7%) | -0.012 | 0.012 |
| Clinically Frailty Scale, median (IQR) | 2.6 (1-4.1) | 3 (2-4) | 0.032 | 0.044 |
| Multidimensional Scale Perceived Social Support, median (IQR) | 60.5 (25.9-96.7) | 72 (59.2-81) | 0.028 | 0.066 |
| AUDIT score, median (IQR) | 0 (0-1.5) | 1 (0-2) | 0.043 | 0.098 |
| Functional Capacity Index, median (IQR) | 1.4 (0.3-3) | 2 (1-3) | 0.001 | 0.101 |
| Charlson Comorbidity Index, median (IQR) | 0.9 (0-2.7) | 1 (0-2.8) | -0.048 | 0.096 |
| Resides at home before admission, n (%) | 95.87 (98.8%) | 97 (99%) | -0.001 | 0.001 |
| Tested positive for COVID-19 during admission, n (%) | 15.7 (16.2%) | 19 (19.4%) | 0.032 | 0.032 |
| Hospital length of stay, days, median (IQR) | 13.5 (6-20.7) | 14 (9.2-19) | -0.018 | 0.100 |

Additional file 1: Table S4: Cause of death at 3-month follow-up.

| Cause | Frequency |
| --- | --- |
| Cancer | 2 |
| Cardiac Arrest | 1 |
| Cardiac Disease | 1 |
| COPD | 2 |
| Unknown | 4 |

Additional file 1: Table S5: Descriptive statistics of secondary outcomes at 3-month follow-up (n = 194).

| Instrument | Scored, n (%) | Score, median (IQR) | Abnormal, n (%) |
| --- | --- | --- | --- |
| ADL dependency | 167 (86.1%) | 0 (0-0) | - |
| IADL dependency | 167 (86.1%) | 0 (0-2) | - |
| EQ5D VAS | 163 (84%) | 75 (50-85) | - |
| EQ5D Utility | 163 (84%) | 0.8 (0.7-1.0) | - |
| HADS Anxiety* | 159 (82%) | 5 (2-8) | 42 (26.4%) |
| HADS Depression* | 160 (82.5%) | 4 (2-7.2) | 40 (25%) |
| IESR* | 159 (82%) | 0.5 (0.2-1.1) | 17 (10.7%) |
| MSPSS* | 155 (79.9%) | 70 (59.5-74) | 41 (26.5%) |
| MoCA* | 141 (72.7%) | 24.5 (20.5-27.3) | 46 (32.6%) |
| * Cutoffs for abnormal scores: HADS >= 8, IESR >= 1.6, MSPSS <= 60 (indicating medium to low perceived support), MoCA >= 27  ADL, activity of daily living; EQ-5D-5L, EuroQoL 5-dimension 5-level; HADS: Hospital Anxiety and Depression Scale; IADL, instrumental activity of daily living; IES-R, impact of event scale – revised; MoCA, Montreal Cognitive Assessment; MSPSS, multidimensional scale of perceived social support | | | |
|  | | | |

## Additional file 1: Table S6: Mortality at 6 months, n=186

| Attribute | Central Tendency (dispersion) |
| --- | --- |
| Death before 6 months, n (%) | 17 (9.1%) |
| Time to death among decedent, days, median (IQR) | 86 (68 to 122) |
|  |  |

Additional file 1: Table S7: Descriptive statistics of secondary outcomes at 6-month follow-up (n = 165).

| Instrument | Scored, n (%) | Score, median (IQR) |
| --- | --- | --- |
| ADL dependency | 165 (98.9%) | 0 (0-0) |
| IADL dependency | 165 (98.9%) | 0 (0-2) |
| EQ5D Visual Analog Scale | 148 (89.1%) | 77.5 (60-85) |
| EQ5D Utility | 149 (89.7%) | 0.8 (0.7-1.0) |

Additional file 1: Table S8: Primary outcome estimates for exposure and covariates.

| Parameter | Risk Ratio | 95% CI | P-value |
| --- | --- | --- | --- |
| Above Median Proportion Unmet Medication Needs |  |  |  |
| Intercept | 0.181 | 0.054 to 0.611 |  |
| High unmet medication needs | 1.252 | 0.749 to 2.094 | 0.391 |
| Number of medication needs | 1.038 | 1.005 to 1.072 |  |
| Age | 1.006 | 0.987 to 1.026 |  |
| Sex: Male | 1.268 | 0.770 to 2.087 |  |
| Study Site: 2 | 0.505 | 0.241 to 1.059 |  |
| Study Site: 3 | 0.320 | 0.180 to 0.568 |  |
| Study Site: 4 | 0.252 | 0.034 to 1.857 |  |
| Study Site: 5 | 0.491 | 0.208 to 1.158 |  |

Additional file 1: Table S9. Primary outcome estimates for exposure constituents and covariates.

| Parameter | Risk Ratio | 95% CI | P value |
| --- | --- | --- | --- |
| 3-Month Mortality |  |  |  |
| Above Median Proportion Unmet Medication Needs | | | |
| Intercept | 0.007 | 0.000 to 0.143 |  |
| High Unmet Medication Needs | 0.132 | 0.018 to 0.992 | 0.049 |
| Number medication needs | 0.999 | 0.838 to 1.192 |  |
| Age | 1.064 | 0.999 to 1.133 |  |
| Sex: Male | 0.838 | 0.218 to 3.219 |  |
| Study Site: 2 | 0.373 | 0.034 to 4.143 |  |
| Study Site: 3 | 0.243 | 0.057 to 1.043 |  |
| Study Site: 4 | 0.000 | 0.000 to 0.000 |  |
| Study Site: 5 | 0.339 | 0.045 to 2.583 |  |
| 3-Month Readmission |  |  |  |
| Above Median Proportion Unmet Medication Needs | | |  |
| Intercept | 0.150 | 0.037 to 0.605 |  |
| High Unmet Medication Needs | 1.717 | 0.96 to 3.068 | 0.068 |
| Number medication needs | 1.046 | 1.013 to 1.08 |  |
| Age | 1.000 | 0.978 to 1.021 |  |
| Sex: Male | 1.256 | 0.737 to 2.141 |  |
| Study Site: 2 | 0.528 | 0.239 to 1.163 |  |
| Study Site: 3 | 0.339 | 0.184 to 0.625 |  |
| Study Site: 4 | 0.364 | 0.053 to 2.516 |  |
| Study Site: 5 | 0.599 | 0.249 to 1.442 |  |

* Death before 3-month follow-up includes 3 participants that also were readmitted before the 3-month follow-up.

Additional file 1: Table S10. Summary of findings for regression models using unmet needs as a continuous variable.

| Parameter | Risk Ratio | 95% CI | Pvalue |
| --- | --- | --- | --- |
| 3-Month Composite Outcome |  |  |  |
| Unmet Medication Needs | 1.207 | 0.180 to 8.108 | 0.847 |
| 3-Month Mortality |  |  |  |
| Unmet Medication Needs | 0.000 | 0.000 to 9.921 | 0.096 |

Additional file 1: Table S11. Sensitivity analysis, includes adjustment for time to assessment of discharge needs follow up.

| Parameter | Risk Ratio | 95% CI | P-value |
| --- | --- | --- | --- |
| 3-Month Mortality |  |  |  |
| High Unmet Medication Needs | 0.140 | 0.018 to 1.092 | 0.061 |
| 3-Month Readmission |  |  |  |
| High Unmet Medication Needs | 1.737 | 0.976 to 3.092 | 0.060 |

Additional file 1: Table S12. Sensitivity analysis, medication needs categorized as all met or 1 or more needs unmet.

| Parameter | Risk Ratio | 95% CI | P-value |
| --- | --- | --- | --- |
| 3-Month Mortality |  |  |  |
| All medication needs met | 3.484 | 1.173 to 10.35 | 0.025 |
| 3-Month Readmission |  |  |  |
| All medication needs met | 0.504 | 0.264 to 0.961 | 0.037 |

1. Sevin CM, Bloom SL, Jackson JC, Wang L, Ely EW, Stollings JL. Comprehensive care of ICU survivors: Development and implementation of an ICU recovery center. *Journal of critical care* 2018; 46: 141-148.

2. Dettling-Ihnenfeldt DS, De Graaff AE, Nollet F, Van Der Schaaf M. Feasibility of Post-Intensive Care Unit Clinics: an observational cohort study of two different approaches. *Minerva anestesiologica* 2015; 81: 865-875.

3. Morandi A, Vasilevskis E, Pandharipande PP, Girard TD, Solberg LM, Neal EB, Koestner T, Torres RE, Thompson JL, Shintani AK, Han JH, Schnelle JF, Fick DM, Ely EW, Kripalani S. Inappropriate medication prescriptions in elderly adults surviving an intensive care unit hospitalization. *J Am Geriatr Soc* 2013; 61: 1128-1134.

4. Stollings JL, Bloom SL, Wang L, Ely EW, Jackson JC, Sevin CM. Critical Care Pharmacists and Medication Management in an ICU Recovery Center. *Ann Pharmacother* 2018; 52: 713-723.
